# Influence of temperature on digital photon counter performance for SPECT

**DOI:** 10.1186/2197-7364-1-S1-A23

**Published:** 2014-07-29

**Authors:** Carmen Bouckaert, Stefaan Vandenberghe, Roel Van Holen

**Affiliations:** MEDISIP, Department of Electronics and Information Systems, Ghent University-iMinds Medical IT-IBiTech, Ghent, Belgium

Digital photon counters (DPC, Philips Digital Photon Counting) are compact photon detectors that are promising for the design of a compact SPECT insert for MRI. Given the small bore of most preclinical MR scanners such an insert has to fulfil strict space constraints.

In DPCs the amount of dark counts increases with temperature and therefore operation at low temperatures is recommended to reduce the effect of dark counts. Compared to the size of the detector, the cooling system is quite bulky and becomes the limiting factor for integration in a compact MRI.

The performance of a DPC-3200-22-44 (32.6x32.6cm2) coupled to a 2mm monolithic LYSO crystal was evaluated at operating temperatures of 18°C and 3°C.

Using a ^57^Co source, a 7% count loss was found when increasing the temperature from 3°C to 18°C. In a next step, the energy resolution was determined using a point grid acquired with a ^99m^Tc beam source. After correcting for the offset caused by the dark counts, the average energy resolution at 3°C and 18°C was calculated to be 32.5% and 33.9%, respectively. The intrinsic spatial resolution was investigated using both the point grid and resolution collimator measurements. Based on the point grid, the average resolution at 3°C and 18°C is 0.48mm and 0.52mm, respectively. This was confirmed using the collimator measurements where the rods with 0.4mm diameter could be discriminated for both temperatures. Finally, the count rate performance, determined by uniformly irradiating the detector, is linear between zero and 20kcps for both temperatures.

These results show that although the performance is somewhat worse at 18°C, the DPC is still suited as a high-resolution detector for SPECT at this temperature. This poses less strict requirements on the cooling, thus obviating the need for a bulky cooling system and enabling SPECT integration in a small animal MRI.

